# MYCs and PIFs Act Independently in Arabidopsis Growth Regulation

**DOI:** 10.1534/g3.120.401188

**Published:** 2020-03-27

**Authors:** Chunmei Li, Kazunari Nozue, Julin N. Maloof

**Affiliations:** * Department of Plant Biology, University of California, Davis, CA 95616; † Rice Research Institute, Sichuan Agricultural University, 211 Huimin Road, Wenjiang District, Chengdu 611130, China; ‡ College of Agriculture and Biology Zhongkai University of Agriculture and Engineering, 24 Dongsha Road, Haizhu District, Guangzhou 510225, China

**Keywords:** MYC2/3/4, PIF4/5/7, growth/defense tradeoff, shade avoidance, RNA-seq

## Abstract

Plants have a variety of strategies to avoid canopy shade and compete with their neighbors for light, collectively called the shade avoidance syndrome (SAS). Plants also have extensive systems to defend themselves against pathogens and herbivores. Defense and shade avoidance are two fundamental components of plant survival and productivity, and there are often tradeoffs between growth and defense. Recently, MYC2, a major positive regulator of defense, was reported to inhibit elongation during shade avoidance. Here, we further investigate the role of MYC2 and the related MYC3 and MYC4 in shade avoidance, and we examine the relationship between MYC2/3/4 and the PIF family of light-regulated transcription factors. We demonstrate that MYC2/3/4 inhibit both elongation and flowering. Furthermore, using both genetic and transcriptomic analysis we find that MYCs and PIFs generally function independently in growth regulation. However, surprisingly, the *pif4/5/7* triple mutant restored the petiole shade avoidance response of *myc2* (*jin1-2*) and *myc2/3/4*. We theorize that increased petiole elongation in *myc2/3/4* could be more due to resource tradeoffs or post-translational modifications rather than interactions with PIF4/5/7 affecting gene regulation.

Plants adapt their growth, physiology, and development to their environment by perceiving abiotic conditions including light, temperature, and nutrient and water availability, as well as biotic conditions including symbiotic, antagonistic, and commensal biota. Plants depend on light for photosynthesis; to optimize light capture, many plants respond to neighbor shade with increased stem and petiole elongation growth (part of the “shade avoidance” syndrome) in order to compete for light (Casal 2013). Defense and shade avoidance are two fundamental components of plant survival and productivity, and there are often tradeoffs between growth and defense (Ballaré 2014). Defense compromised mutants show an increased growth rate (Abreu and Munné-Bosch 2009; Züst *et al.* 2011) while plants with chemically or genetically activated defense pathways have reduced growth (Heidel *et al.* 2004; van Hulten *et al.* 2006). Furthermore, a plant defense hormone, jasmonic acid (JA), influences growth (Noir *et al.* 2013; Attaran *et al.* 2014). Shade avoidance, brought about by dense planting, has been shown to reduce agricultural yields, an effect attributed to changes in carbon allocation that favor stem elongation over seed, fruit, or tuber production (Boccalandro *et al.* 2003; Chincinska *et al.* 2008). There is extensive cross-talk between the defense and growth pathways, and generally plants prioritize growth over defense when faced with neighbor shade (Ballaré 2014). Recently, interactions between key components in growth and immunity signaling pathways have been found to be important for controlling these growth/defense trade-offs (de Wit *et al.* 2013; Ballaré 2014; De Bruyne *et al.* 2014; Huot *et al.* 2014; Nozue *et al.* 2018). Many unanswered questions remain about the mechanisms underlying growth/defense interactions. Investigating and understanding the mechanism of growth/defense trade-off under shade will help to develop strategies for maximizing yield in dense agricultural plantings.

Current knowledge of how plants undergoing shade avoidance prioritize growth over defense is limited. Previous studies have focused on how shade limits defense signaling (Ballaré 2014); Shade reduces plant immunity through interactions with two central plant defense hormones pathways, JA (Moreno *et al.* 2009; de Wit *et al.* 2013) and SA. Resistance against a hemi-biotrophic (*Pseudomonas syringae* pv *tomato*, *Pst*) and a necrotrophic (*Botrytis cinerea*) pathogen is suppressed by shade treatment. Further, shade has been shown to reduce downstream transcriptional responsiveness to JA and SA. Less is understood about how defense pathways regulate growth. In a study of shade avoidance signaling components in adult Arabidopsis, a new link between defense and shade was defined: the JA pathway is important not only for regulating defense but also regulating elongation. Furthermore, growth/defense trade-offs were shown to be uncoupled in the *jaz10 phyB* and *jazQ phyB* mutants that showed both robust growth and heightened anti-insect defense (Campos *et al.* 2016). This uncoupling was attributed, at least in part, to parallel activation of MYC and Phytochrome-Interacting Factor (PIF) transcription factors that are repressed by JAZ and phyB in wild-type plants, respectively (Campos *et al.* 2016; Cerrudo *et al.* 2017). The current model of JA/growth interactions focuses on the GA signaling inhibitory DELLA proteins as regulators of the growth/defense trade-off (Leone *et al.* 2014). The model posits that under sun conditions DELLAs bind the growth-promoting PIF proteins (thereby inhibiting growth) and bind the JA signaling repressor JAZ proteins (allowing increased JA to elicit a defense response). In shade conditions DELLAs are degraded, releasing PIFs to promote growth and releasing JAZs to inhibit defense. JA also increases DELLA accumulation, suggesting that JA could inhibit growth via DELLAs. JAZ proteins repress MYC2 (Chini *et al.* 2009), MYC3, and MYC4 transcription factors (Niu *et al.* 2011; Fernández-Calvo *et al.* 2011). In the presence of JA, JAZ proteins are degraded allowing MYC2/3/4 to alter transcription of JA regulated genes (Chini *et al.* 2009; Niu *et al.* 2011; Fernández-Calvo *et al.* 2011). Under shade or in *phyB* mutants, MYC2/3/4 are destabilized and suggest that this destabilization is critical for proper shade avoidance growth (Chico *et al.* 2014). *myc2* knock-out mutants have been found to have constitutively elongated petioles and therefore do not exhibit petiole shade avoidance (Nozue *et al.* 2015). Thus wild-type MYC2 functions not only to promote defense but also to inhibit growth.

Previous studies revealed the inhibition role of MYC2 in growth during shade avoidance (Nozue *et al.* 2015) and that shade-mediated accumulation of PIF proteins and the subsequent increase in auxin biosynthesis and signaling are critical for shade avoidance (Lorrain *et al.* 2008; Nozue *et al.* 2011; Li *et al.* 2012; Hornitschek *et al.* 2012). Since PIF proteins promote growth (Paik *et al.* 2017), MYC2 inhibits growth (Nozue *et al.* 2015), and the PAIR database (Lin *et al.* 2011) predicts an interaction between MYC2 and PIF4, we hypothesized that MYC2 could inhibit growth by repressing PIF function. In defense signaling, MYC3 and MYC4 heterodimerize with MYC2 and MYC2/3/4 are partially redundant in promoting immunity (Fernández-Calvo *et al.* 2011), therefore it is possible that this redundancy may also apply to growth inhibition. To address how MYC2 inhibits growth, in this paper, we analyzed shade avoidance phenotypes of the *myc2* single (*jin1-2*) *myc3/4* double, *myc2/3/4* triple, *myc2pif4/5/7* quadruple, and *myc2/3/4pif4/5/7* sextuple mutants grown in simulated sun and shade. We found that MYC2/MYC3/MYC4 function redundantly in growth inhibition, and that MYCs and PIFs function in parallel to regulate growth in high red/far-red (R/FR) light conditions. Surprisingly, the *pif4/5/7* triple mutant restored the shade avoidance response of *jin1-2* and *myc2/3/4*. RNAseq revealed that a number of genes controlling flowering time were enriched in *myc2/3/4* triple mutant, identified tradeoffs between indole-glucosinolate (indoleGS) and indole acetic acid (IAA; auxin) as a possible mechanism for enhanced elongation in *myc2/3/4*, and were consistent with independent action of MYC2/3/4 and PIF4/5/7. We concluded that *myc2/3/4* increased growth could be more due to resource tradeoffs than gene regulation.

## Materials And Methods

### Growth conditions

For simulated sun condition, white light (cool-white fluorescent light) was supplemented with far-red light provided by LEDs (Orbitec, inc) to obtain R/FR = 2.7 (high red/far-red, high R/FR). For simulated shade condition, white light was supplemented with far-red LEDS to obtain R/FR between 0.3 and 0.4 (low R/FR). Both conditions had 80–120 μE of Photosynthetically Active Radiation (PAR). Plants were grown under long day condition (16 hr light/8 hr dark) at constant temperature (22°). Ambient light spectrum was measured by Black-Comet (StellarNet, Florida)(Nozue *et al.* 2015).

### Plant materials

Arabidopsis seeds: Mutant seeds in the Col-0 background (*jin1-2*) (Lorenzo *et al.* 2004), *myc2* (jin1-2) *myc3* (445B11 GABI-KAT) *myc4* (GK 491E10) were obtained from Roberto Solano (Campus Universidad Autónoma), and *pif4* (*pif4-101*, Garlic_114_G06) *pif5* (*pil6-1*, SALK_087012) *pif7* (*pif7-1*) were obtained from Christian Fankhauser (University of Lausanne). *jin1-2 pif4/5/7* and *myc2/3/4pif4/5/7* were generated by crossing *myc2/3/4* with *pif4/5/7* and genotyping F2 and F3 generation to recover homozygous plants. Primers used in genotyping are listed in Supplementary Table 1. For phenotyping, Arabidopsis seeds were imbibed with water in 1.5 mL tubes and stored under dark at 4° for four days. Five seeds were transferred to soil in a 4x9 well flat and placed under simulated sun condition. 7 days after sowing, excess seedlings were removed to leave one well-grown plant per pot, and 13 days after sowing, the plants were either transferred to shade or kept in the sun condition. Genotype positions were randomized in each replicate set.

### RNA-seq library preparation and sequencing

For RNA extraction plants were grown for 13 days under high R/FR until they had 2 cotyledons and 2 expanded true leaves. Half of these plants were treated with shade starting at ZT 6 and the remainder were left in the sun. We prepared four replicates of each sample at 1 hr and 49 hr after sun and shade treatment and three plants were pooled for each replicate. When collecting samples at 1h treatment, cotyledons, leaves, hypocotyls, and roots were removed, leaving apical tissue for samples. When collecting samples at 49h, only apical tissues, and the leaves and petioles newly elongated after treatment (leaves 3 and 4) were harvested for samples. Total RNA from the plants was extracted using Trizol (MacRae 2007). Two to five μg of total RNA was used to construct mRNA library using NEBNext Poly(A) mRNA Magnetic Isolation Module (NEB). cDNA libraries were made by using Strand-Specific mRNA-library prep kit for Illumina sequencing (Amaryllis Nucleics). The resulting cDNA libraries were sequenced by HiSeq4000 with 50 bp single end mode (the DNA Technologies and Expression Analysis Cores at the UC Davis Genome Center, supported by NIH Shared Instrumentation Grant 1S10OD010786-01).

### Differential expression analysis and over-representation analysis (ORA)

Reads were sorted according to barcodes and filtered to remove adaptor contamination by custom Perl scripts (https://github.com/MaloofLab/SAS_defense_transcriptome). Reads were trimmed by Trimomatic (Bolger *et al.* 2014) and mapped by Kallisto (Bray *et al.* 2016) to Arabidopsis TAIR10 cDNA sequences (Supplementary Table S2). Differentially expressed genes were extracted by edgeR package in R statistical environment (FDR < 0.05). ORA was done by GOseq package (Young *et al.* 2010) in R statistical environment. GO analysis was done by using GO category database package from Bioconductor org.At.tair.db (Carlson *et al.*) and ANNOTATE package (Gentleman *et al.* 2004). For ORA of hormone responsive genes custom categories were used as in (Nozue *et al.* 2015, 2018).

### Phenotype measurement and analysis

For scoring leaf phenotypes, 26 day old plants were dissected and leaf images were recorded by a flatbed scanner (Perfection V800 Photo, Epson), and scanned images were measured using ImageJ and the LeafJ plugin as described by Maloof *et al.* and analyzed as described by Nozue *et al.* (2015) to determine petiole length, leaf blade length, leaf blade width, and leaf blade area. Days to bolting was used as a measurement of flowering time.

Leaf phenotypes (petiole length, leaf blade length, leaf blade width, leaf blade area) and flowering time (days to bolting) were measured from 3 sets of experiments. Each phenotype was fitted by lme4 (Bates *et al.* 2014) and lmerTest (Kuznetsova *et al.* 2017) packages in R, using a model such as:

trait∼genotype+treatment+genotype:treatment+(treatment|set)+ε

where genotype represent a genotypic line (wildtype or different mutants), treatment is sun or shade condition, genotype:treatment is interaction of “genotype” and “treatment”, (treatment|set) is the random effect associated with the treatment in set of experiments, and ε is the error. The model was applied to each trait to calculate coefficient (“sun” value). For leaf traits where we measured across multiple leaves (from leaf 5 to leaf 8) for a given trait we treated leaf as a random effect, using the following model

trait=genotype+treatment+genotype:treatment+(1|leaf)+(treatment|set)+ε

Mutants were considered to have a defect in SAS when the genotype:treatment term was significant (*P* < 0.05), indicating that the genotype of the plant (mutant *vs.* wild-type) altered the response to shade. Bootstrap resampling was used to calculate 95% confidence intervals for plotting.

### Data availability

Strains are available upon request. Sequence data are available at the NCBI Short Read Archive under accession number PRJNA512107. Analysis scripts are deposited in github repositories: https://github.com/MaloofLab/SAS_defense_phenotyping, and https://github.com/MaloofLab/SAS_defense_transcriptome. Supplemental material available at figshare: https://doi.org/10.25387/g3.9544832.

## Results And Discussion

### MYC2/MYC3/MYC4 function in growth inhibition

**Figure 1:**
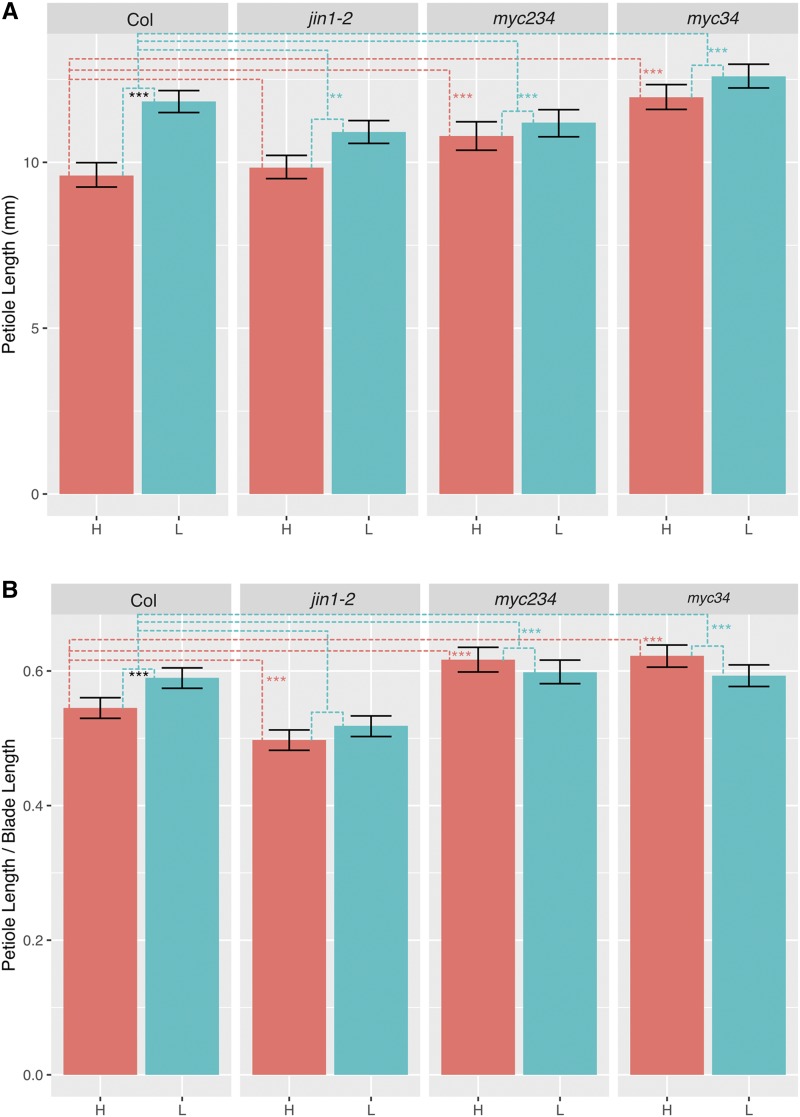
Petiole phenotype of *jin1-2*, *myc3/4* and *myc2/3/4* in high R/FR (“H”) and low R/FR (“L”). (A) Petiole length. (B) Petiole/Blade length ratio. Significant differences were evaluated using a linear mixed-effects model with genotype, treatment, and the genotype-by-treatment interaction as fixed effects. Black asterisks indicate difference between Col in high R/FR and Col in low R/FR. Dashed lines indicate other comparisons being made. Red dashed lines show comparisons between mutants and Col in high R/FR and red asterisks indicate significant differences from Col. Blue dashed lines show comparisons between mutants and Col for the response to low R/FR (low R/FR – high R/FR) and blue asterisks indicate significant differences from Col. The fifth to ninth leaves were measured from 8 to 10 plants per genotype/condition from 3 independent experimental trials. Error bars show 95% bootstrap confidence interval. * *P* < 0.05; ** *P* < 0.01; *** *P* < 0.001.

MYC2 is a basic helix-loop-helix (bHLH) TF important for JA mediated immune responses that acts semi-redundantly with its homologs, MYC3 and MYC4 . *myc2* mutants show partially reduced SAS in adult plants, raising the possibility that the MYC2/3/4 redundancy is also true for SAS (Nozue *et al.* 2015). Therefore, we hypothesized that MYC2/3/4 function redundantly in growth inhibition in the same way as in plant defense. To test this hypothesis, we analyzed the *myc3/4* double and *myc2/3/4* triple mutant for shade avoidance. To induce shade avoidance responses, we used supplemental far red (FR) LEDs to lower the red/far-red ratio to ∼0.3 (low R/FR) from the control value of 2.7 (high R/FR). The double mutant, *myc3/4*, and the triple mutant, *myc2/3/4*, showed a constitutive “shade” phenotype with increased shade avoidance elongation in two indexes (petiole length or petiole length/blade length ratio) under both high and low R/FR treatment compared to Col (Figure 1). Both *myc3/4* and *myc2/3/4* mutants displayed increased elongation in high R:FR compared to *jin1-2*, which is a point mutation mutant of MYC2, indicating that MYC2/3/4, not only function redundantly in plant defense, but also in growth inhibition.

### Flowering time of MYC2/3/4

**Figure 2:**
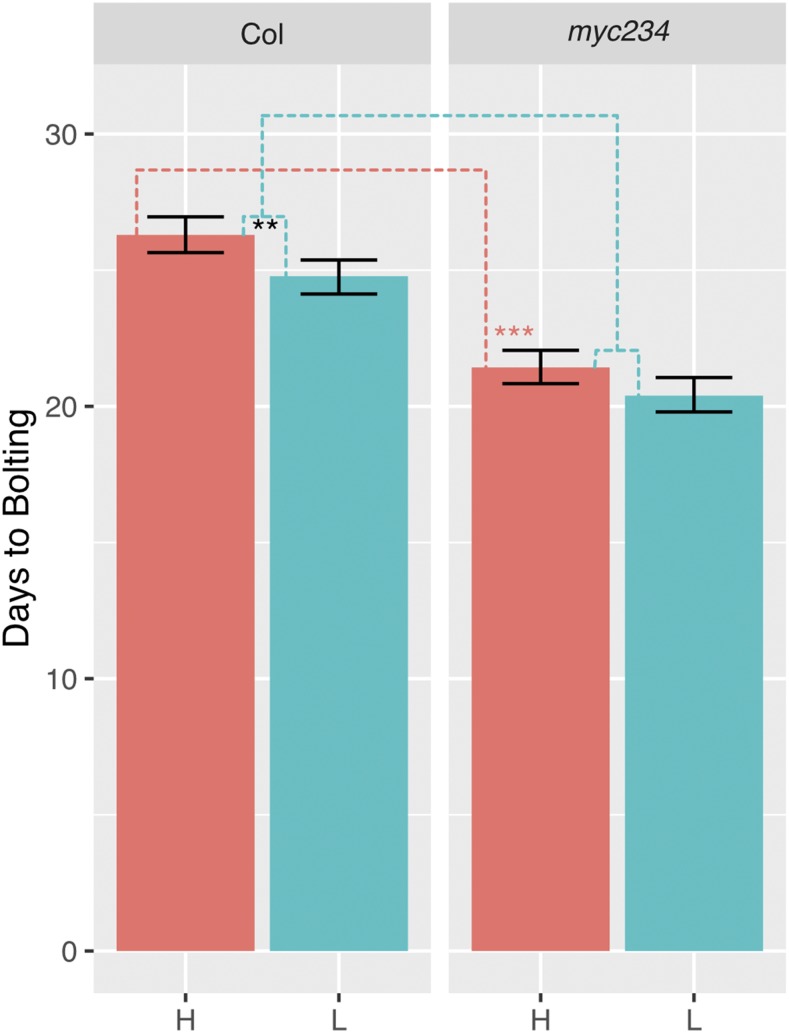
Flowering time of *myc2/3/4*. Significant differences were evaluated using a linear mixed-effects model with genotype, treatment, and the genotype-by-treatment interaction as fixed effects. “H” denotes high R/FR and “L” denotes low R/FR. Black asterisks indicate difference between Col in high R/FR and Col in low R/FR. Dashed lines indicate other comparisons being made. Red dashed lines show comparisons between mutant and Col in high R/FR and red asterisks indicate significant differences from Col. Blue dashed lines show comparisons between mutant and Col for the response to low R/FR (low R/FR – high R/FR) and blue asterisks indicate significant differences from Col (in this case there was no significant difference in response to low R/FR between Col and *myc2/3/4)*. Flowering time is days to bolting from 9 to 12 plants per genotype/condition from 2 independent experimental trials. Error bars show 95% bootstrap confidence interval. * *P* < 0.05; ** *P* < 0.01; *** *P* < 0.001.

In addition to hypocotyl and petiole elongation, acceleration of flowering time is another aspect of the shade avoidance syndrome. To ask if *myc2/3/4* solely affected petiole elongation or instead altered other shade-regulated phenotypes, we measured days to bolting in *myc2/3/4* and wild type plants as a measure of flowering time. The *myc2/3/4* mutant exhibited significantly earlier flowering phenotype under high R/FR condition, compared to the wild type (Figure 2). As expected, low R/FR treatment caused a significant acceleration of flowering for wild type Col plants as compared to high R/FR. We found that low R/FR also accelerated flowering *myc2/3/4*, and that the magnitude of this effect was similar to the effect of low R/FR on wild type. Thus, while *myc2/3/4* mutations do affect flowering time, this effect is independent of low R/FR.

### MYCs and PIFs act in parallel to regulate petiole growth in high R/FR

**Figure 3:**
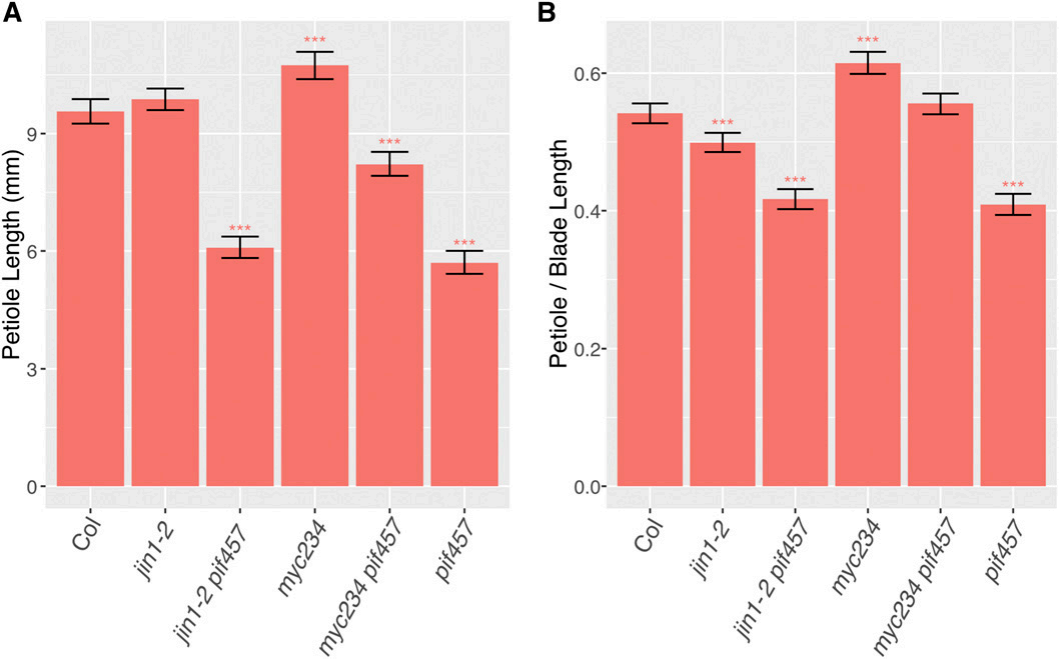
Petiole length of *myc* and *pif* mutants in high R/FR. (A) Petiole length. (B) Petiole/Blade length ratio. Significant differences were evaluated using a linear mixed-effects model with genotype, treatment, and the genotype-by-treatment interaction as fixed effects. Asterisks indicate significant differences from Col in high R/FR. The fifth to ninth leaves were measured from 8 to 10 plants per genotype/condition from 3 independent experimental trials. Error bars show 95% bootstrap confidence interval. * *P* < 0.05; ** *P* < 0.01; *** *P* < 0.001.

Having established that MYCs inhibit petiole elongation, we hypothesized that MYCs may inhibit growth by repressing PIFs function. This idea is based on the fact that PIF proteins accumulate under shade increasing auxin biosynthesis and signaling pathway which are critical for shade avoidance elongation (Lorrain *et al.* 2008; Nozue *et al.* 2011; Hornitschek *et al.* 2012; Leivar and Monte 2014). Since the *myc* mutants show a constitutive shade phenotype we reasoned that if PIFs are required for the mutant *myc* phenotype that *pif* mutants should be epistatic to *myc* mutants in high R/FR conditions. Therefore, we created strains that combined mutations in the three *PIF* genes critical for shade regulated elongation with various *myc* mutants. Specifically, we constructed a *jin1-2/pif4/5/7* quadruple mutant strain and a *myc2/3/4pif4/5/7* sextuple mutant strain. In high R/FR, the *MYC* and PIF genes appear to act additively (Figure 3). On their own, the *myc* mutants are longer than wildtype and the *pif* triple mutant is shorter. The *myc*, *pif* quadruple and sextuple mutants have intermediate phenotypes. For example, the sextuple mutant was longer than *pif4/5/7*, but shorter than *myc2/3/4*, suggesting that MYC2/3/4 and PIF4/5/7 regulate petiole elongation through parallel pathways.

### pif4/5/7 rescues the shade avoidance response in myc2/3/4

**Figure 4:**
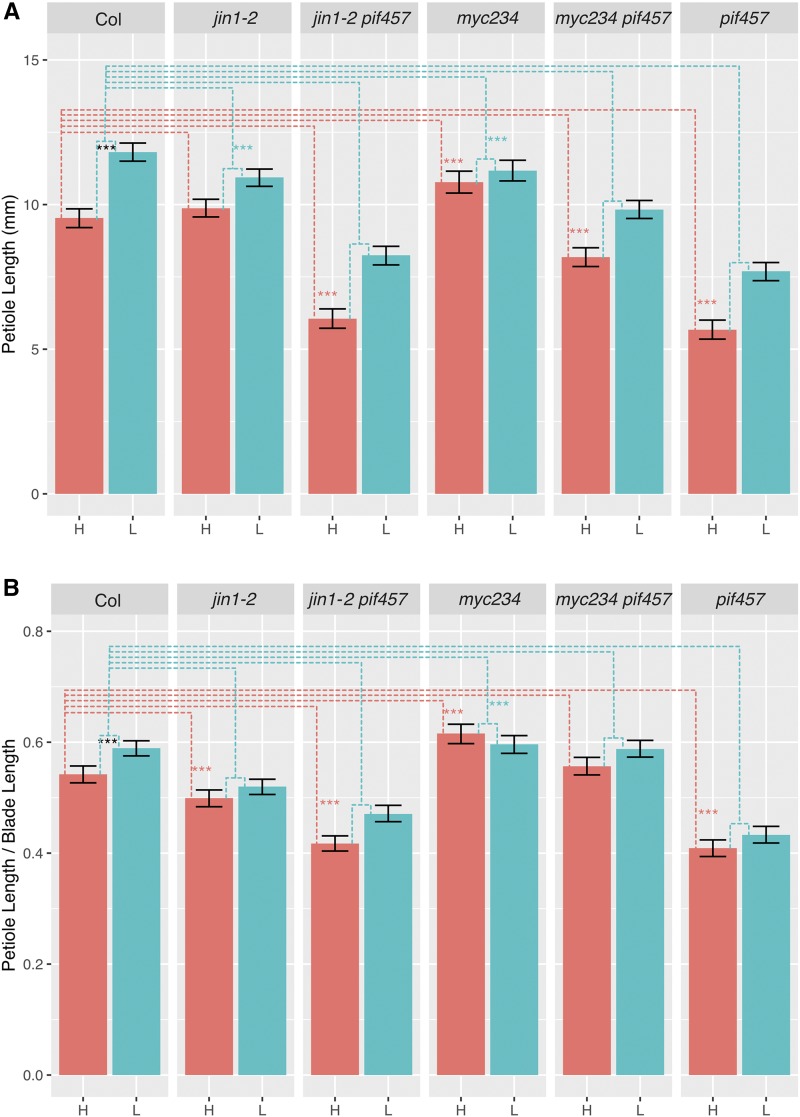
Petiole length of *myc* and *pif* mutants in high and low R/FR. (A) Petiole length. (B) Petiole/Blade length ratio. “H” denotes high R/FR and “L” denotes low R/FR. Significant differences were evaluated using a linear mixed-effects model with genotype, treatment, and the genotype-by-treatment interaction as fixed effects. Black asterisks indicate difference between Col in high R/FR and Col in low R/FR. Dashed lines indicate other comparisons being made. Red dashed lines show comparisons between mutants and Col in high R/FR and red asterisks indicate significant differences from Col. Blue dashed lines show comparisons between mutants and Col for the response to low R/FR (low R/FR – high R/FR) and blue asterisks indicate significant differences from Col. The fifth to ninth leaves were measured from 8 to 10 plants per genotype/condition from 3 independent experimental trials. Error bars show 95% bootstrap confidence interval. * *P* < 0.05; ** *P* < 0.01; *** *P* < 0.001.

Under low R/FR conditions *pif4/5/7* mutant plants had shorter petioles than wild type, as we had also observed in high R/FR (Figure 3, 4). However, the *pif4/5/7* petiole elongation *response* to low R/FR was comparable to the wild type Col response, indicating that, surprisingly, the petiole shade avoidance response still exists in the *pif4/5/7* mutant (Figure 4). *pif4/5/7* mutants have been previously reported to elongate petioles in response to low R/FR, however, with a reduced response that we did not observe in our study (de Wit *et al.* 2015); we (Nozue *et al.* 2015) also previously reported a reduced response of *pif4/5* mutant petioles to low R/FR. We have verified the genotype of the *pif4/5/7* plants used in the current study; the discrepancies between this study and previous reports likely indicate sensitivity to undetermined environmental factors for this phenotype.

The p*if/myc* quadruple and sextuple mutants also showed an interesting phenotype under low R/FR. Specifically, we found that *pif4/5/7* rescued the petiole shade avoidance response of both *jin1-2* and *myc2/3/4*. That is, both *jin1-2/pif4/5/7* and *myc2/3/4pif4/5/7* showed a petiole elongation shade response indistinguishable from wild type and *pif4/5/7*. One possible explanation is that petiole length could already be near its maximum in the *jin1-2* and *myc2/3/4* mutants such that it is not physically possible for these plants to respond to the shade cue with additional elongation. Under this scenario, removing *PIF4/5/7* function shortens the *myc* mutant petioles such that they are below their physical limit and can elongate in response to the low R/FR cue.

### Transcriptome Analysis of myc2/3/4 mutants

**Figure 5:**
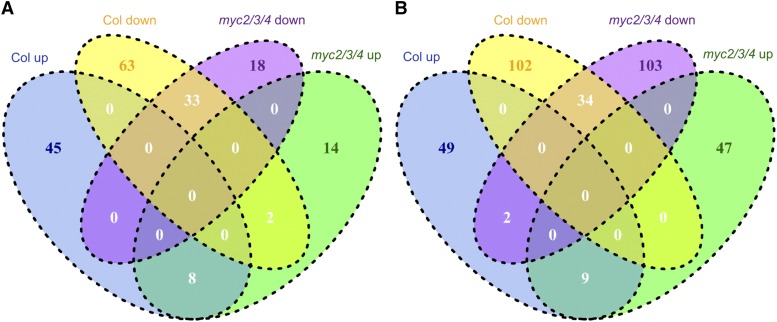
Differentially expressed genes in Col and *myc2/3/4* under different experimental conditions. (A) Differentially expressed genes at 1h high R/FR and low R/FR treatments. (B) Differentially expressed genes at 49h high R/FR and low R/FR treatments. “Col up” and “Col down” indicate genes up-regulated and down-regulated, respectively, in Col under low R/FR. “*myc2/3/4* up” and “*myc2/3/4* down” indicate genes up-regulated and down-regulated, respectively, in *myc2/3/4* under low R/FR. All genes are differentially expressed at *P* < 0.05. For differentially expressed genes in each genotype, the corresponding genotype under high R/FR condition was used as reference.

**Figure 6:**
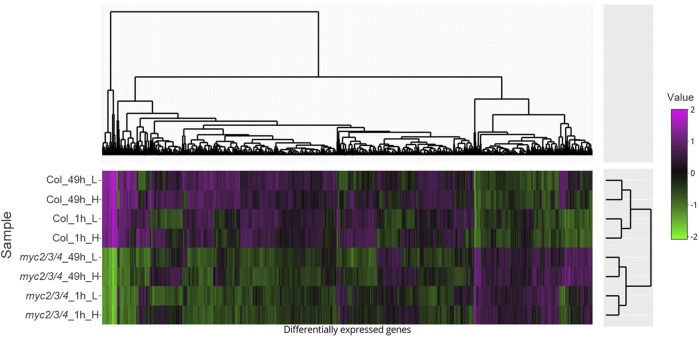
Heatmap of differentially expressed genes in Col and *myc2/3/4* under different experimental conditions. The differentially expressed genes in *myc2/3/4* include all genes that are differentially expressed between high and low R/FR within genotype, and between Col and *myc2/3/4* at *P* < 0.05.

Overall, our genetic experiments suggested that MYCs and PIFs regulate elongation via parallel pathways. To test this finding at the molecular level and to identify possible direct and indirect downstream targets of MYC2/3/4 related to elongation, we performed RNA-seq to compare the transcript profile of wild type and *myc2/3/4* at 1 h and 49 h treatment in high R/FR and low R/FR. The number of differentially expressed genes (DEGs; Table 1), the overlap between them (Figure 5), and the clustering of the DEGs (Figure 6) are summarized and discussed in detail below.

**Table 1:** Summary of low R/FR responsive genes in Col and *myc2/3/4*

GENOTYPE	TIME POINT	NO. OF UP-REGULATED GENES	NO. OF DOWN-REGULATED GENES
Col	1 h	53	98
Col	49 h	60	136
*myc2/3/4*	1 h	24	51
*myc2/3/4*	49 h	56	139

**Figure 7:**
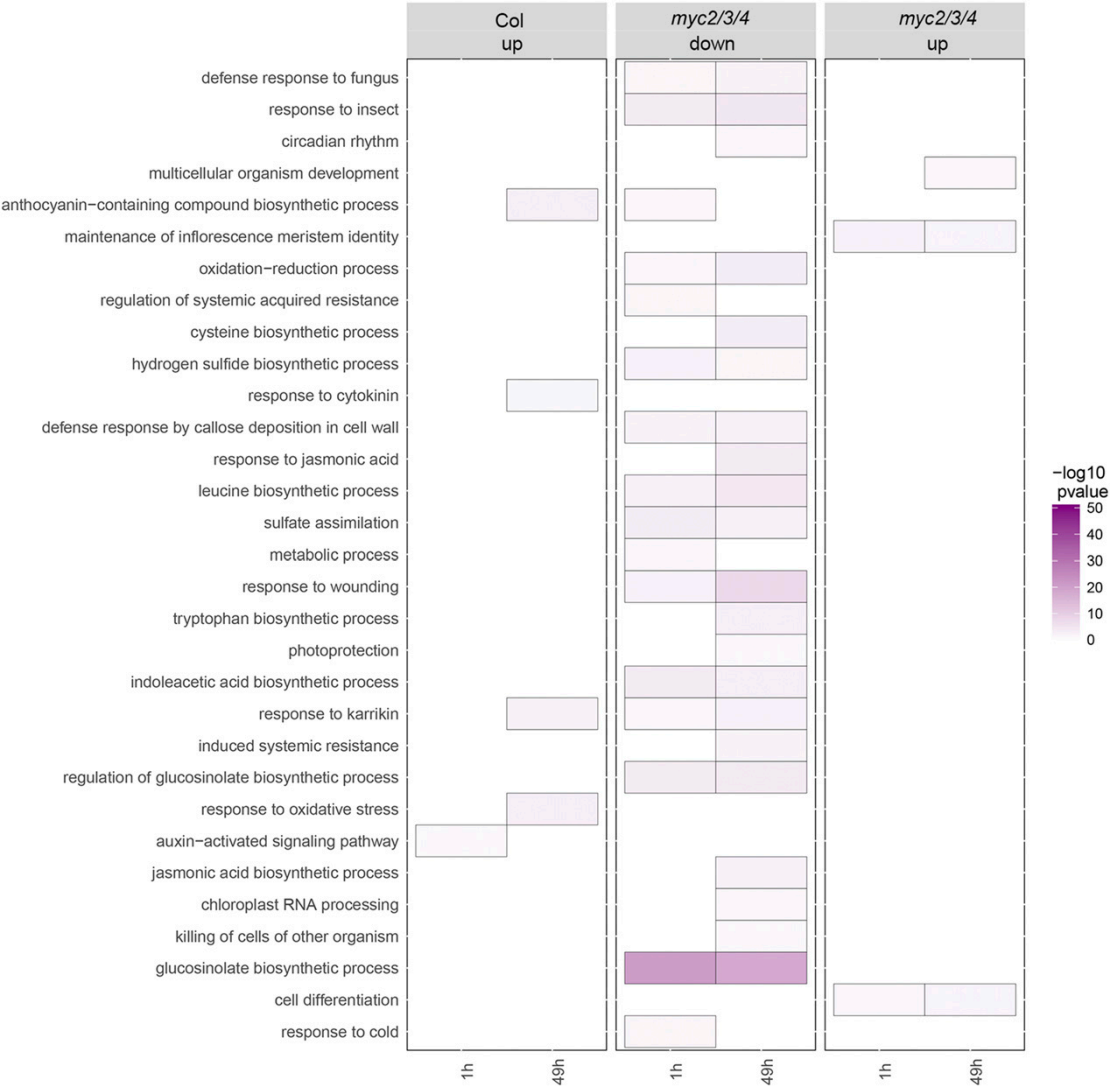
Over-represented GO term in differentially expressed genes in Col and *myc2/3/4* under different experimental conditions. For each GO term, all genes enriched at *P* < 0.05. “Col up” column indicates the over-represented GO terms in up-regulated genes compared to Col under high R/FR. “*myc2/3/4* up” and “*myc2/3/4* down” columns indicate the over-represented GO terms in up- and down-regulated genes, respectively, compared to Col.

In the differentially expressed gene set of wild type Col, auxin-activated signaling pathway genes were up regulated in Col after 1h of low R/FR treatment, including known shade-responsive genes, *IAA19*, *IAA29*, *ATHB2*, matching expectation and showing that our low R/FR treatment worked (Table 1; Supplementary Table S3). Previous work has shown that glucosinolate (GS) biosynthesis genes are direct targets of MYC2/3/4 in defense signaling (Schweizer *et al.* 2013). We found that most of these genes were down regulated in *myc2/3/4* in 2-week-old plants (our experiment) as well, even though the previous study used leaves of 4-week-old plants (Figure 7; Supplementary Table S4), consistent with results that MYC2/3/4 directly activates transcription of GS biosynthesis genes. Thus, overall treatment and genotype effects are as expected.

### Flowering related genes are enriched in myc2/3/4 differentially expressed genes

In accordance with the early-flowering phenotype of *myc2/3/4*, we also found that a number of flowering related genes were up regulated in *myc2/3/4* at both 1 and 49 h treatment, compared to the wild-type Col. These genes had not previously been identified as being up regulated in *myc2/3/4* mutants, likely because the previous study harvested 4-week-old plants that had finished bolting. Up regulated genes include *SOC1* (*SUPPRESSOR OF OVEREXPRESSION OF CONSTANS1*)*/AGL 20* (*AGAMOUS LIKE 20*) (Borner *et al.* 2000; Onouchi *et al.* 2000; Samach *et al.* 2000; Lee *et al.* 2000) which is an integrator in flower development, *AGL24* (*AGAMOUS‐LIKE 24*) (Michaels *et al.* 2003; Fujita *et al.* 2003) and *LFY* (LEAFY)(Weigel *et al.* 1992), which act downstream of *SOC1* and are positively regulated by *SOC1* (Samach *et al.* 2000; Lee *et al.* 2000, 2008; Moon *et al.* 2003; Liu *et al.* 2007, 2008), *AP1*(*APETALA1*) (Mandel *et al.* 1992), which is positively regulated by *LFY* (Wagner *et al.* 1999) and negatively regulates *AGL24* (Yu *et al.* 2004), and *FUL* (*FRUITFUL*)(Gu *et al.* 1998), which is the downstream of *FT* (*FLOWERING LOCUS T*) and is positively regulated by *FT* (Teper-Bamnolker and Samach 2005) (Figure 2; Figure 7; Supplementary Table S4, S5, S6). MYC2/3/4 have been demonstrated to inhibit flowering by repressing *FT* (Wang *et al.* 2017). *FT* was not detected to be differentially expressed in our condition (Supplementary table S4, S5, S6, S7, S8). The reason could be that the expression of *FT* is induced by CO (CONSTANS) protein, which only accumulates in the late afternoon in long day (Samach *et al.* 2000), while the tissue was collected at ZT7 in our experiment when *FT* might not be expressed. Overall, the flowering genes that we detected as being upregulated in *myc2/3/4* mutants are consistent with induction through FT and support the findings of Wang *et al.* 2017.

### Transcriptome analysis reveals a possible mechanism for increased elongation in myc2/3/4

**Figure 8:**
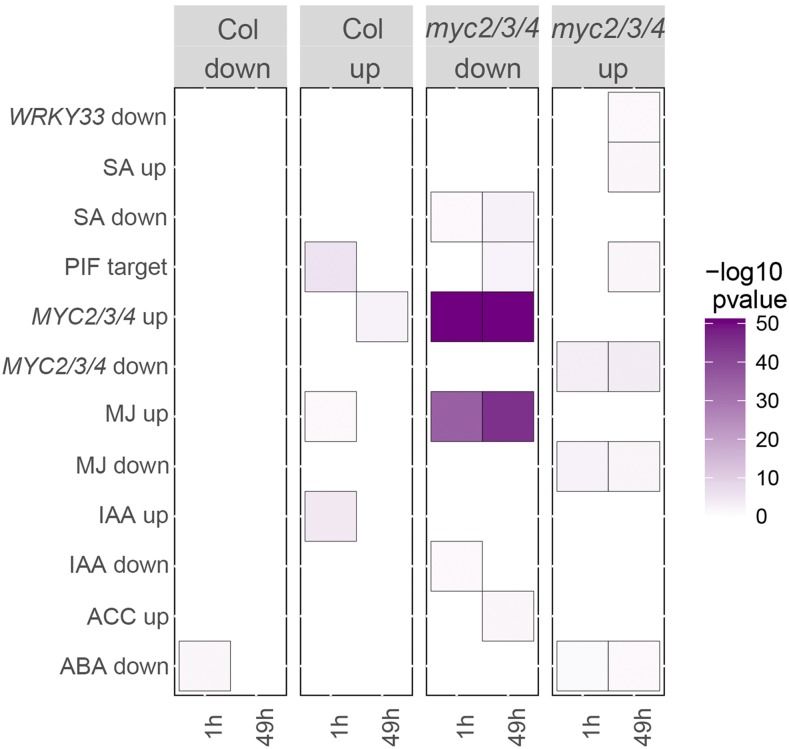
Over-represented custom categories in differentially expressed genes in Col and *myc2/3/4* under different experimental condition. For each custom category, all genes enriched at *P* < 0.05. “Col up” and “Col down” columns indicate the over-represented custom category in up- and down-regulated genes, respectively, compared to Col under high R/FR. “*myc2/3/4* up” and “*myc2/3/4* down” columns indicate the over-represented custom category in up- and down-regulated genes, respectively, compared to Col. ABA up or ABA down, abscisic acid up- or down-regulated; ACC up or ACC down, 1-aminocyclopropane-1-carboxylic acid up- or downregulated; IAA up or IAA down, indole-3-acetic acid (IAA) up- or downregulated; MJ up or MJ down, methyl jasmonate (MJ) up- or down-regulated; *MYC2/3/4* up or *MYC2/3/4* down, up- or down-regulated by MYC234 genes; PIF target, PIF target genes; SA up or SA down, salicylic acid up- or down-regulated.

Because of the role of *MYC2/3/4* in inhibiting petiole elongation, we expected growth-related genes, such as auxin (indoleacetic acid, IAA) biosynthetic genes and IAA-regulated genes, to be upregulated in the *myc2/3/4* mutant. However, contrary to our expectation, IAA biosynthetic genes including *SUR1*, *SUR2*, *CYP79B2*/*3* and *TRP2* were down regulated in *myc2/3/4*. Since MYC2/3/4 regulate glucosinolate biosynthesis, one explanation for our finding is the known relationship between indole-GS and auxin biosynthesis. Specifically, indole-GS contributes to auxin biosynthesis via the metabolic intermediates indole-3-acetaldoxime (IAOx) and indole-3-acetonitrile (IAN). *CYP79B2/B3* are involved in formation of IAOx from TRP, *SUR1* and *SUR2* are involved in the biosynthesis of indole-GS from IAOx, and indole-GS can be digested by myrosinases to form IAN (Halkier and Gershenzon 2006; Malka and Cheng 2017). It has been reported that inactivation of GS biosynthesis genes acting post IAOx, such as *SUR1*, *SUR2* and *UGT74B1*, leads to elevated IAA level along with impaired indole GS (Delarue *et al.* 1998; Bak *et al.* 2001; Malka and Cheng 2017). Thus, SUR1 and SUR2 balance GS and IAA biosynthesis. *SUR1* and *SUR2* were down-regulated in our study in the *myc2/3/4* triple mutant, and furthermore, the results showed that the known genes down regulated by IAA including *CYP79B2/3* and *GSTF11* were down-regulated in our study (Figure 8; Supplementary Table S9). Previous studies have shown that MYC2 binds to the *SUR2* promoter (Schweizer *et al.* 2013) and have shown that low R/FR reduces the *SUR2* reaction product I3M in a *JAZ10* dependent manner (Cargnel *et al.* 2014) suggesting that low R/FR inactivates GS synthesis via JAZ / MYC2 interactions (Cargnel *et al.* 2014). In addition, *myc2* mutants have been found to have increased auxin content (Huang *et al.* 2017). Therefore, it is possible that free IAA level also increases in *myc2/3/4* mutant and leads to petiole elongation in the mutant. Genes classified as up-regulated by IAA were not differentially expressed in our experiment, however most such genes are only transiently activated during shade avoidance (Nozue 2018), so would not serve as good indicators of long-term IAA growth promotion. In summary, the RNAseq results point to an increase of IAA via GS/IAA tradeoffs as a possible explanation for increased elongation in the *myc2/3/4* mutants.

Besides the possible increase of IAA in *myc2/3/4*, MYC2 has been reported to suppress the activity of COP1 (CONSTITUTIVE PHOTOMORPHOGENIC 1) in promoting HY5 (ELONGATED HYPOCOTYL 5) degradation and was found to be required for COP1 suppression of hypocotyl elongation in JA signaling pathway (Zheng *et al.* 2017). In our RNA-seq data, *COP1* was up-regulated in *myc2/3/4* at both 1h and 49h treatment (Supplementary Table S5, S6). It is possible that MYC2/3/4 inhibits petiole growth in adult plants through COP1. However, HY5 only accumulates in plants younger than 7-days old (Hardtke *et al.* 2000), so this mechanisms seems unlikely. It is possible that MYC2/3/4 affect petiole elongation by inhibiting the activity of COP1 in degrading other targets, such as HYH (HY5-HOMOLOG) (Holm *et al.* 2002).

Consistent with the idea that MYCs and PIFs act independently in growth regulation, PIF genes were not differentially expressed in *myc2/3/4*. We also examined whether there was overlap between PIF target genes and genes differentially expressed in *myc2/3/4*. Since PIF genes promote elongation and elongation is also promoted in *myc2/3/4* mutants, if PIFs and MYCs regulate a common growth pathway we would expect positive PIF targets to also be upregulated in *myc2/3/4*. We tested this idea and found that while 6 of 39 positive PIF targets were differentially expressed in *myc2/3/4* relative to Col (significant overlap, *P* = 0.002), these genes were down-regulated in *myc2/3/4* and/or less induced by low R/FR (Supplementary Figure S1). Similarly, three of ten negative PIF targets were differentially expressed in *myc2/3/4*, but two of these three were upregulated. Since the PIF targets are regulated opposite of expectation in *myc2/3/4* this data supports the conclusion that the *myc2/3/4* petiole growth phenotype occurs independently of PIF4/5/7 action. The opposite regulation may indicate negative feedback through the PIF pathway.

## Conclusions

Our genetic experiments show that PIF4/5/7 and MYC2/3/4 act additively to control petiole growth in high R/FR but that in low R/FR, PIF4/5/7 function are required for the *myc2/3/4* constitutive petiole shade avoidance phenotype. Our RNA-seq experiments support the hypothesis that PIF4/5/7 and MYC2/3/4 can regulate growth independently. We propose at least three possible mechanisms for MYC2/3/4 regulation of petiole growth that are not mutually exclusive. One possibility is that the *myc2/3/4* increased growth is more due to energetic tradeoffs rather than gene regulation. The second possibility is that impaired GS biosynthesis in *myc2/3/4* mutants elevates IAA levels. Third, the loss of MYC2/3/4 function in the triple mutant could relieve its inhibition on COP1 activity, leading to increased growth.
